# Sialylation and Galectin-3 in Microglia-Mediated Neuroinflammation and Neurodegeneration

**DOI:** 10.3389/fncel.2020.00162

**Published:** 2020-06-09

**Authors:** Mar Puigdellívol, David H. Allendorf, Guy C. Brown

**Affiliations:** Department of Biochemistry, University of Cambridge, Cambridge, United Kingdom

**Keywords:** sialic acid, desialylation, galectin-3, phagocytosis, microglia, neurodegeneration, aging

## Abstract

Microglia are brain macrophages that mediate neuroinflammation and contribute to and protect against neurodegeneration. The terminal sugar residue of all glycoproteins and glycolipids on the surface of mammalian cells is normally sialic acid, and addition of this negatively charged residue is known as “sialylation,” whereas removal by sialidases is known as “desialylation.” High sialylation of the neuronal cell surface inhibits microglial phagocytosis of such neurons, via: (i) activating sialic acid receptors (Siglecs) on microglia that inhibit phagocytosis and (ii) inhibiting binding of opsonins C1q, C3, and galectin-3. Microglial sialylation inhibits inflammatory activation of microglia via: (i) activating Siglec receptors CD22 and CD33 on microglia that inhibit phagocytosis and (ii) inhibiting Toll-like receptor 4 (TLR4), complement receptor 3 (CR3), and other microglial receptors. When activated, microglia release a sialidase activity that desialylates both microglia and neurons, activating the microglia and rendering the neurons susceptible to phagocytosis. Activated microglia also release galectin-3 (Gal-3), which: (i) further activates microglia via binding to TLR4 and TREM2, (ii) binds to desialylated neurons opsonizing them for phagocytosis via Mer tyrosine kinase, and (iii) promotes Aβ aggregation and toxicity *in vivo*. Gal-3 and desialylation may increase in a variety of brain pathologies. Thus, Gal-3 and sialidases are potential treatment targets to prevent neuroinflammation and neurodegeneration.

## Introduction

Microglia are brain macrophages that mediate neuroinflammation and can protect against neurodegeneration, for example, by removing protein aggregates, phagocytosing debris, and aiding repair. However, microglia can in some circumstances also contribute to neurodegeneration, for example, by mediating chronic neuroinflammation, or by excessive phagocytosis of synapses or neurons. This article reviews the roles of sialylation and galectin-3 (Gal-3) in microglia-mediated neuroinflammation and neurodegeneration. That means we will be reviewing the effects of changes in (a) sialylation of brain cells and (b) extracellular Gal-3 on microglial activation and neurodegeneration. In general, Gal-3 is upregulated and released by microglia during neuroinflammation and promotes neuroinflammation and phagocytosis (Shin, 2013; Chen et al., 2014). Conversely, sialylation of microglia and neurons inhibits neuroinflammation and phagocytosis, in part by blocking Gal-3 binding. Moreover, neuroinflammation can promote desialylation.

Microglia constantly survey the brain with their long and rapidly moving processes, looking for signs of pathogens, damage, or protein aggregates. If they detect such signs they become “activated,” i.e., they retract their processes, express inflammatory genes, produce reactive oxygen species, release chemokines and pro-inflammatory cytokines, upregulate phagocytosis, and may migrate toward the pathogens, damage, or aggregates. All of this helps the microglia phagocytose the pathogens, damage, or aggregates, and thereby remove the problem. However, if for whatever reason the pathogens, damage or aggregates are not effectively removed, then the microglia may become chronically activated, and this may result in damage to neurons, due to, for example, excessive cytokine production, excessive reactive oxygen species production, or excessive phagocytosis of synapses and neurons. Thus, chronic neuroinflammation may contribute to neurodegeneration (Ransohoff, 2016; Nichols et al., 2019).

## Sialic Acid: Structure, Function, and Signaling

**FIGURE 1:**
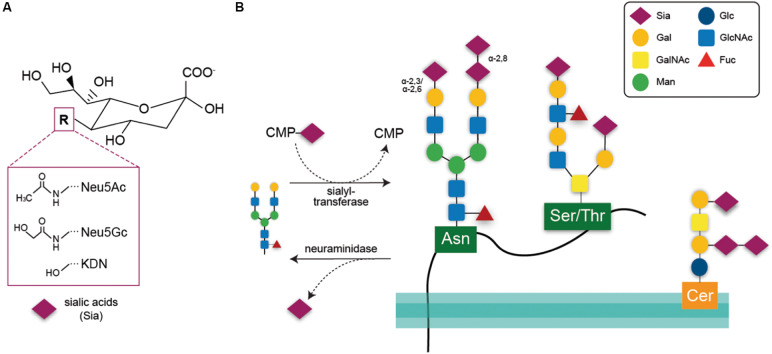
**(A)** Chemical structure of the three core sialic acids, *N*-acetylneuraminic acid (Neu5Ac), *N*-glycolylneuraminic acid (Neu5Gc), and deaminoneuraminic acid (KDN). These core structures may be further modified, e.g., by sulfation or methylation. **(B)** Sialic acids are transferred onto acceptor glycans via sialyl-transferase enzymes in the Golgi that use the activated cytidine-5’-monophosphate-sialic acid (CMP-Sia) as a donor molecule. Sialyl residues may be added terminally to galactose residues in α-2,3 or α-2,6 linkage or to sialic acid residues in α-2,8 linkage. Such glycan chains may be attached to glycoproteins via asparagine residue (*N*-glycan) or to serine or threonine residues (*O*-glycan). Glycosylation of lipids is exemplified here by a ganglioside composed of ceramide (Cer) to an oligosaccharide chain. Sialic acids may be released via hydrolytic enzymes termed neuraminidases. Gal, galactose; GalNAc, *N*-acetylgalactosamine; Man, mannose; Glc, glucose; GlcNAc, *N*-acetylglucosamine; Fuc, fucose.

The terms sialylation and desialylation refer, respectively, to the addition and removal of the sugar sialic acid on the non-reducing termini of oligosaccharide chains attached to glycoproteins or glycolipids. In vertebrates, sialic acids are a heterogeneous family of nine-carbon monosaccharides with core structures consisting of either *N*-acetylneuraminic acid (Neu5Ac), *N*-glycolylneuraminic acid (Neu5Gc), or deaminoneuraminic acid (Kdn) (Figure 1A). In the human brain, the main sialic acid core structure is Neu5Ac, whereas levels of Neu5Gc and Kdn are very low (Chou et al., 1998; Inoue and Kitajima, 2006; Davies and Varki, 2015). The sialic acid core structures may be further modified by methylation, acetylation, and sulfation at the fourth, seventh, eighth, and ninth positions, generating more than 50 sialic acid species (Angata and Varki, 2002). Sialic acids are synthesized *de-novo* using the precursor *N*-acetylmannosamine-6-phosphate (ManNAc-6-P). ManNAc-6-P is the product of glucosamine (UDP-*N*-acetyl)-2-epimerase/*N*-acetylmannosamine kinase (GNE), which is the rate-limiting enzyme for sialic acid synthesis (Keppler et al., 1999). After synthesis, sialic acid is activated by conversion to CMP-sialic acid by the nuclear enzyme CMP-sialic acid synthetase (CMAS) and then transported into the Golgi where it acts as a substrate for sialyl-transferases (STs; Munster-Kuhnel et al., 2004).

Within the Golgi, sialic acids are attached to the sugars of glycolipids or glycoproteins (Figure 1B) via ST enzymes. In humans, 20 different enzymes transfer sialic acids to galactose (Gal) or *N*-acetylgalactosamine (GalNAc) acceptor sugars via α-2,3- or α-2,6-bonds (enzymes: ST3Gal, ST6Gal, ST6GalNAc) or to other sialic acid moieties via α-2,8 bonds (ST8Sia) (reviewed by Harduin-Lepers et al., 2001).

A small subset of glycoproteins, including particularly neural cell adhesion molecule (NCAM) are polysialylated, i.e., carry linear chains of 50–150 sialic acid residues linked α2,8, added by polysialyltransferases in the Golgi. Polysialylation is particularly abundant in the brain, and regulates cell adhesion, synaptogenesis, memory, and neurogenesis, as well as binding neurotrophins, growth factors, and neurotransmitters (Sato and Kitajima, 2013; Colley et al., 2014). In mouse brain, polysialylation dramatically decreases 2 weeks after birth, and almost disappears by 8 weeks, except in olfactory bulb, hippocampus, amygdala, suprachiasmatic nucleus, and prefrontal cortex (Abe et al., 2019). Polysialylated NCAM is also present on the surface of microglia, and rapidly decreases in response to LPS activation as a result of the microglial release of sialidase, which then desialylates NCAM (Sumida et al., 2015). In contrast, polysialylated neuropilin-2 is normally present within the microglial Golgi, but is rapidly released to the surface by LPS (Werneburg et al., 2015).

Gangliosides are lipids composed of a glycosphingolipid (ceramide and oligosaccharide) with one or more sialic acids. About 60 different gangliosides are known, which differ mainly in the position and number of sialic acid residues. The most abundant ganglioside in the brain is GM1, which is neuroprotective in multiple brain pathologies (Magistretti et al., 2019).

Sialic acid residues are negatively charged and are major contributors to the charge and hydrophilicity of the cellular surface, which reduces interactions and adhesion between cells (Varki, 1997; Byrne et al., 2007). Moreover, the presence of sialic acid on a glycoprotein or glycolipid may modulate cell signaling: sialic acids themselves serve as ligands for lectins such as selectins and sialic acid-binding immunoglobulin-type lectins (Siglecs). Siglecs form a family of transmembrane proteins that contain an extracellular carbohydrate recognition domain (CRD) that can bind sialic acid residues of the same or different cells, and a cytoplasmic tail that (in most Siglecs) contains an immunoreceptor tyrosine-based inhibition motif (ITIM) domain. Upon binding of the CRD to sialyl residues, the ITIM domain is phosphorylated and recruits and activates protein tyrosine phosphatases, such as Src homology domain-containing phosphatase-1 (SHP-1). These phosphatases reverse the tyrosine phosphorylation of signaling proteins, such as Syk, induced by activating receptors. Thus, Siglecs and the sialylation state of self- or target cells’ glycocalyx act as important negative regulators of cellular activation and phagocytosis (Varki and Angata, 2006). Siglecs are typically expressed by hematopoietic cells (Von and Bochner, 2008) and, in the CNS, are mainly expressed by microglia. Structure, function, and signaling properties of microglial Siglecs (Siglec-1, Siglec-2/CD22, Siglec-3/CD33, Siglec-4/MAG, Siglec-11, and Siglec-E/F/H) have been extensively reviewed elsewhere (Linnartz-Gerlach et al., 2014; Siddiqui et al., 2019).

Some Siglecs have been subject to rapid evolution, and are poorly conserved, potentially resulting in divergent functions. For example, human and mouse CD33 have limited homology in their intracellular domains, such that human CD33 has an ITIM and an ITIM-like domain, whereas mouse CD33 only has an ITIM-like domain (Brinkman-Van der Linden et al., 2003). Thus, human CD33 has been found to inhibit monocyte and microglial phagocytosis, whereas mouse CD33 has been reported to have no effect on phagocytosis (Bhattacherjee et al., 2019).

Desialylation describes the removal of sialyl-residues from glycoconjugates, which is generally mediated by hydrolytic enzymes called sialidases or neuraminidases (Neu) (these terms are synonymous) (Wei and Wang, 2019). Mammalian cells express four different sialidases, Neu1-4. Neu1 is found either in the lysosome or on the surface of plasma membrane and is one of the main enzymes degrading sialo-glycoproteins. Neu1 has the highest expression of the sialidases in human tissue, and may cleave sialic acids linked in α-2,3 and—to a lesser degree—α-2,6 and α-2,8 (Miyagi and Tsuiki, 1984). The cytosolic Neu2 sialidase is expressed at a very low level in humans, and cleaves sialic acids from glycoproteins and glycolipids with a similar linkage-specificity to Neu1 (Miyagi and Tsuiki, 1985). Neu3 is found on the plasma membrane, and has the highest specificity for sialylated gangliosides, cleaving α-2,3, α-2,6, and α-2,8 linked sialic acids equally. Neu4 is found on internal membranes, and has preference for gangliosides (Miyagi and Yamaguchi, 2012; Pshezhetsky and Ashmarina, 2018). Notably, loss of function mutations or knock out of sialidase enzymes gives rise to a variety of brain pathologies in humans and mice. Thus, mutations in the human neuraminidase 1 gene cause sialidosis characterized by the accumulation of sialylated glycans in lysosomes. Brain pathologies associated with this severe lysosomal storage disorder are ataxia, mental retardation, and seizures (Seyrantepe et al., 2003). Double knockout of Neu3 and Neu4 in mice caused accumulation of the GM3 ganglioside inside CNS cells, such as microglia and neurons, causing neuroinflammation, impairment of neurite formation, and memory loss (Pan et al., 2017).

Sialidases are important for degradation of glycoproteins and glycolipids, as well as the recycling of sialic acid. However, the removal of sialic acid residues from the cell surface by sialidases may also trigger cell signaling events as this desialylation: (i) reduces Siglec signaling, (ii) activates other receptors, (iii) decreases some gangliosides and increases others, and (iv) changes binding of lectins, opsonins, and complement. Multiple receptors, such as Toll-like receptor 4 (TLR4), can be activated by desialylation (Pshezhetsky and Ashmarina, 2013). Some lectins, such as Gal-3, are released by inflammatory activated microglia and bind to *N*-acetyl-lactosamine residues of glycoproteins only when terminal sialic acid residues are removed (Nomura et al., 2017). The removal of sialic acids from the neuronal cell surface encourages binding of the classical complement cascade proteins C1q and C3, which are important opsonins tagging cells for phagocytosis (Linnartz et al., 2012). Inhibition of complement by cell surface sialylation may be mainly mediated by complement factor H, a serum protein recruited to cells by binding sialic acid residues. When recruited by cell surface sialylation, factor H acts as a negative regulator of complement depositions by promoting degradation of C3 convertase and already deposited C3b (Blaum et al., 2015). Mutations in the factor H sialyl-recognition domain can cause atypical hemolytic uremic syndrome (aHUS), a disorder characterized by increased C3b deposition and lysis of blood cells (Hyvarinen et al., 2016) indicating that the factor H-sialic acid interaction is a key regulator of the alternative pathway of complement activation. Therefore, desialylation may promote opsonization of cells by increased binding and decreased degradation of classical complement proteins.

In the following sections, we will use the terms “sialylated” or “desialylated” in a simplified manner to describe the presence or absence, respectively, of sialyl residues on surface glycans, irrespective of linkage or modifications.

## Effects of Desialylation/Sialylation in the Brain

Sialic acids are particularly abundant in the brain, including within neuronal gangliosides and as polysialic acid on NCAM (Finne et al., 1983; Pan et al., 2005). Polysialylation of NCAM on neurons regulates neurite outgrowth (Landmesser et al., 1990), axon pathfinding (Tang et al., 1994), synaptogenesis (Dityatev et al., 2004), and long-term potentiation (LTP) in the hippocampus (Becker et al., 1996; Muller et al., 1996; Senkov et al., 2012; Hildebrandt and Dityatev, 2015). Acute stress has been shown to rapidly decrease polysialylation in olfactory bulb and prefrontal cortex in mice, apparently due to sialidases from microglia and astrocytes (Abe et al., 2019). Neural activity has been shown to rapidly increase neuronal (and astrocytic) surface sialidase activity, causing neuronal desialylation, which in turn modifies memory formation (Minami et al., 2016, 2017). Thus, rapid changes in cell-surface sialylation may occur physiologically, usually as a result of transfer of Neu1 or Neu4 to the cell surface, and more dramatic changes may occur as a result of neuroinflammation (see below).

Although sialylation has been found to be dispensable for germ layer formation and early development of the embryo (Abeln et al., 2017), several studies have shown the relevance of sialylation during mammalian development, as demonstrated by the fact that homozygous knockout of either (1) GNE, necessary for sialic acid biosynthesis (Schwarzkopf et al., 2002) or (2) CMAS, necessary for sialic acid activation (Abeln et al., 2019) is embryonically lethal in mice. Interestingly, the phenotype exhibited by CMAS knockout mice was rescued by depleting maternal component C3, indicating that sialylation protects the embryo against attack by maternal complement (Abeln et al., 2019).

**FIGURE 2:**
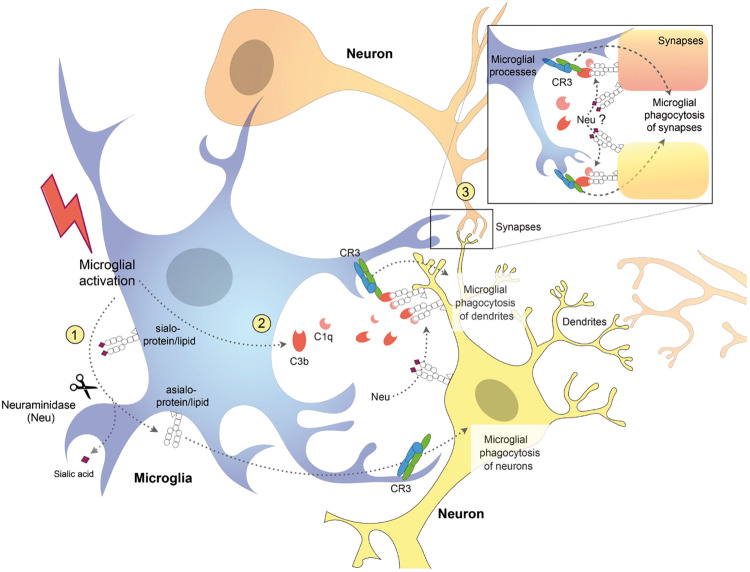
Schematic diagram showing potential mechanisms for complement receptor 3 (CR3)-dependent microglial phagocytosis of neurons, dendrites, and synapses. Activated microglia (1) desialylate their surface via neuraminidase (Neu) which stimulates microglial phagocytosis of neurons via CR3 (Allendorf et al., 2020b) and (2) release complement proteins C1q and C3b, which opsonize desialylated neuronal dendrites and (3) synapses, stimulating their phagocytosis via microglial CR3 (Linnartz et al., 2012). Neuraminidase released from microglia or onto the surface of neurons, desialylates the neuronal surface, and promotes binding of C1q and C3b, stimulating microglial phagocytosis of neurons, dendrites, and synapses.

In contrast to homozygous GNE knockout mice, heterozygous GNE knockout is not lethal in mice, and reduces sialylation by roughly 50%, and so can be used to investigate the effects of reduced sialylation in mice (Klaus et al., 2020). These mice display microglial activation and neuronal synaptic loss, followed by a slow increase in age-related and complement-dependent neuronal loss, indicating a protective role of sialic acids against microglial phagocytosis and physiological aging in the mouse brain (Klaus et al., 2020). Interestingly, loss of synapses precedes neuronal loss in several neurodegenerative diseases (Fricker et al., 2018; Rajendran and Paolicelli, 2018) and complement-mediated microglial phagocytosis of synapses is aberrantly activated and contributes to synaptic loss in mouse models of Alzheimer’s disease (AD) (Stevens et al., 2007; Schafer and Stevens, 2010; Hong et al., 2016). However, these studies did not determine how complement is binding to synapses during development or neurodegeneration. One intriguing possibility is that complement is binding to synapses because they are desialylated (Figure 2). Indeed, it has been shown that desialylation of neurons facilitates C1q and C3b binding detected by complement receptor 3 (CR3) on microglia surface, which leads to phagocytosis of neuronal dendrites (Linnartz et al., 2012). Thus, there is *in vitro* and *in vivo* evidence that desialylation of neurons or neuronal parts can cause complement-mediated microglial phagocytosis of those neurons, synapses, or dendrites.

We have recently found that different stimuli, including LPS, fibrillar amyloid beta (Aβ) and TAU, induced desialylation of the microglial surface (Allendorf et al., 2020b). This desialylation of microglia in turn enhanced microglial phagocytosis via activating CR3, and induced microglia to phagocytose healthy neurons (Figure 2). Addition of LPS or Aβ to glial-neuronal cultures induced neuronal loss that could be blocked by inhibiting sialidases or CR3 (Allendorf et al., 2020b). This suggests that inflammatory stimuli can induce desialylation of microglia, which enhances phagocytosis that may contribute to neurodegeneration. Recent studies suggest that removal of sialyl residues from the microglial cell surface may also activate TLR-mediated signaling. Intracerebral injection of microbial sialidase caused microglial TLR4 and TLR2 activation *in vivo* (Fernandez-Arjona et al., 2019) and *in vitro* (Fernandez-Arjona et al., 2019; Allendorf et al., 2020a). Moreover, we found in the BV-2 microglial cell line that LPS causes Neu1 to translocate to the cell surface, where it desialylates TLR4, which enhances and prolongs microglial activation (Allendorf et al., 2020a). We previously reported that LPS-activated BV2 microglia released a sialidase activity that could desialylate neighboring cells (Nomura et al., 2017). Similarly, Sumida et al. (2015) reported that in the Ra2 microglial cell line, LPS caused a rapid and reversible release of a sialidase activity on exovesicles, which removed polysialic acids from the microglial surface. These studies suggest that activated microglia have the potential to desialylate both themselves and surrounding neurons. *In vivo*, it was shown that LPS injection into the corpus callosum of rat pups induced a dramatic increase in neuraminidase activity (Neu1 and Neu4) leading to a persistent desialylation of glycoproteins and neurons (Demina et al., 2018). Although the authors found that the brain distributions of microglia and neuraminidase activity were different, this does not rule out that neuraminidase activity is being released from microglia (or other cells). Importantly, what this work shows is that neuroinflammation (induced by LPS) can cause desialylation of the brain.

## Sialyltransferases in Neuropathology

In humans, mutations in the ganglioside-specific sialyltransferase ST3Gal5 (GM3 synthase) gene cause: infantile-onset epilepsy syndrome (Simpson et al., 2004) and salt and pepper syndrome, with severe intellectual disability (Boccuto et al., 2014). A mutation in another ganglioside-selective biosynthetic gene, B4GALNT1 (GM2 synthase), result in the presence of progressive motor neuropathy accompanied also by cognitive deficits (Harlalka et al., 2013). Consistent with human studies, mice lacking GM2 synthase have been found to develop progressive motor deficits (Chiavegatto et al., 2000). Mutations in the sialyltransferase ST3Gal3 cause: non-syndromic autosomal recessive intellectual disability (Hu et al., 2011) or West syndrome, an age-dependent epileptic encephalopathic syndrome (Edvardson et al., 2013). ST3Gal2/3 double-null mice had decreased myelin, reduced neuronal marker expression, abnormal dendrites, and exhibited cognitive and motor coordination deficits (Yoo et al., 2015). Mice lacking complex gangliosides also exhibited demyelination and axonal degeneration (Sheikh et al., 1999). Interestingly, neuronal, but not glial, expression of complex gangliosides was sufficient to prevent age-dependent degenerative phenotype in mice (Yao et al., 2014). This all suggests that gangliosides maintain myelin and axons but is also compatible with the idea of sialylation preventing microglial activation and microglial phagocytosis of neurons and synapses (Figure 2).

GWAS and other studies have found a positive association between polysialyltransferases and schizophrenia (Maziade et al., 2005; Arai et al., 2006; Tao et al., 2007), psychotic and mood disorders (McAuley et al., 2012), and autism spectrum disorders (Zhiling et al., 2008; Anney et al., 2010). Changes in polysialylation of NCAM1 may also contribute to Parkinson’s disease and AD (Mikkonen et al., 1999; Oizumi et al., 2008; Murray et al., 2016, 2018). A variety of mechanisms by which changes in polysialic acid may contribute to disease have been suggested, including changes in cell–cell interactions, ion channels, neurotrophins (BDNF, NT3, and NGF), neurotransmitters, and growth factors (Sato and Kitajima, 2013). However, the findings are also compatible with the idea that polysialylation inhibits microglial activation and microglial phagocytosis of neurons and synapses, and the reduced polysialylation seen in these neuropathologies induces excessive microglial activation and phagocytosis.

## Siglec Receptors in Pathological Processes

Sialic acid-binding Ig-like lectin (Siglec) receptors have been linked to neurodegenerative and aging processes (extensively reviewed in Siddiqui et al., 2019; Duan and Paulson, 2020). Siglec receptors on microglia can be activated by sialic acid residues present on the neuronal surface, which inhibit microglial phagocytosis, while desialylation of neurons leads to microglia phagocytosis of the desialylated neurons or dendrites. One abundant inhibitory Siglec receptor expressed on microglia of the human brain is Siglec-11 (Angata et al., 2002) which has been found to inhibit microglia neurotoxicity upon interaction with sialic acids on the neuronal glycocalyx (Linnartz et al., 2010). Siglec-11 expression in microglia suppressed cytokine release and microglial phagocytosis of polysialylated neurons and neurites (Wang and Neumann, 2010). Interestingly, although both microglia and neurons expressed polysialylated NCAM, only the latter appeared to activate the Siglec-11-mediated neuroprotection.

In mice, Siglec-E recognizes sialic acid residues on the neuronal glycocalyx and has been shown to act as a negative regulator of phagocytosis of neuronal debris and the associated production of superoxide radicals (Claude et al., 2013).

Human microglia abundantly express another inhibitory Siglec receptor, Siglec-3, also called CD33. Several GWAS studies have indicated that CD33 is a risk factor for AD (Bertram et al., 2008; Hollingworth et al., 2011; Naj et al., 2011; Walker et al., 2015). The normal, full-length form of CD33 inhibits microglial phagocytosis of Aβ, while the short form of CD33, lacking exon 2 encoding the sialic acid ligand-binding domain, does not inhibit phagocytosis of Aβ, and may thereby reduce AD risk (Bradshaw et al., 2013; Griciuc et al., 2013; Malik et al., 2013; Raj et al., 2014; Siddiqui et al., 2017; Estus et al., 2019). CD33 is thought to be activated by sialic acid residues on the same cell rather than adjacent cells, so desialylation of microglia may activate microglial phagocytosis in part by removing the CD33 block on phagocytosis.

Interestingly, Siglec-2, also known as CD22, has also been identified as a negative regulator of microglial phagocytosis in the aging brain (Pluvinage et al., 2019). Knockout of CD22 in BV-2 microglia increased phagocytosis, and microglial sialylation inhibited phagocytosis partly by activating CD22 (Pluvinage et al., 2019). Importantly, the authors found that inhibition of CD22 on aged microglia *in vivo* facilitates the clearance of myelin debris, amyloid-β oligomers, and α-synuclein fibrils. Long-term inhibition of CD22 partially restores the transcriptional state of aged microglia to a younger homeostatic state and improves cognitive function in aged mice. Importantly, CD22 is upregulated not only in aging brains but also in brains of AD (Friedman et al., 2018) amyotrophic lateral sclerosis (Funikov et al., 2018), and Niemann–Pick type C (Cougnoux et al., 2018). Thus, CD22, as well as CD33 and Siglec-11, are potential therapeutic targets to modify neuroinflammation and neurodegeneration.

Importantly, most human Siglecs have undergone rapid, recent evolution, such that there are no clear orthologs between humans and mice, and there are significant differences in ligand specificity (Linnartz-Gerlach et al., 2014). Moreover, while the above Siglec receptors (Siglec-11, CD33) are abundantly expressed on human microglia, mouse microglia abundantly express others, including CD33-related Siglec-E and CD22 (extensively reviewed in Duan and Paulson, 2020). Thus, using mouse models to study the roles of Siglec receptors in human physiology or disease is not always appropriate. Nonetheless, the above studies confirm the functional role of sialic acids in the brain and encourage future studies aiming to investigate the potential of modulating Siglec expression/function on microglia as new therapeutic strategies to delay or prevent neurodegeneration and age-dependent cognitive deficits.

## Galectin-3

**FIGURE 3:**
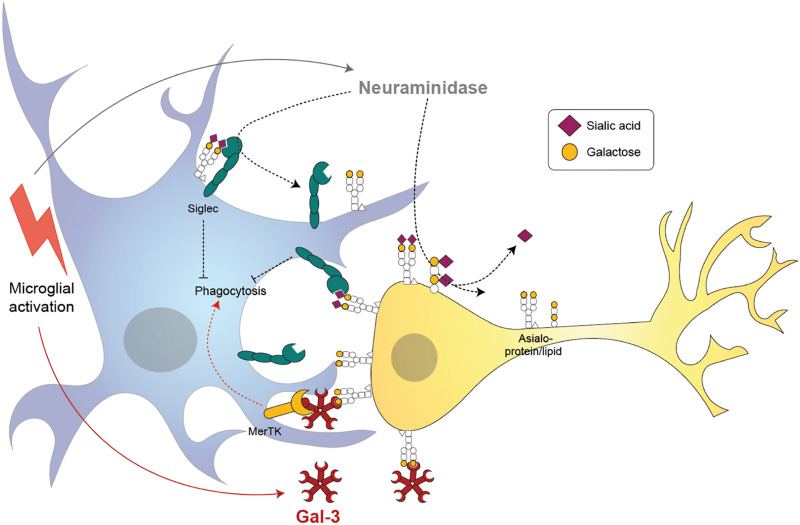
Proposed mechanisms of microglia-induced neurophagy via galectin-3 (Gal-3) and desialylation of microglial and neuronal receptors. Microglia release a neuraminidase after inflammatory activation (i.e., by lipopolysaccharide) that removes sialyl residues on the microglia and surrounding neurons. Desialylation reduces binding of sialic acid binding immunoglobuline-like receptors (Siglecs) in *cis* and *trans*, thus potentially increasing microglial phagocytosis. Activated microglia release the soluble lectin Gal-3 which binds to the penultimate sugar on glycan chains, galactose. Gal-3 opsonizes desialylated neurons and induces phagocytosis by microglia via Mer tyrosine kinase (MerTK).

Galectin-3 is one of the 14 known mammalian galectins, which are lectins (sugar-binding proteins) binding to galactose residues. Gal-3 has a C-terminal carbohydrate-recognition domain that preferentially binds to *N*-acetyl-lactosamine (a disaccharide of galactose and *N*-acetyl-glucosamine) residues in glycoproteins or glycolipids (Hirabayashi et al., 2002). Gal-3 is normally a soluble monomer, but when the C-terminal binds *N*-acetyl-lactosamine on glycoproteins or glycolipids, then the N-terminal oligomerizes with the N-terminal of other Gal-3 units bound to sugars, to form pentamers cross-linking the glycoproteins or glycolipids (Ahmad et al., 2004). Oligomerization of Gal-3 appears to be mediated by liquid-liquid phase separation of aromatic residues in the N-terminal (Chiu et al., 2020). *N*-acetyl-lactosamine is found in the sugar chains of most glycoproteins or glycolipids; however, binding is normally blocked by a terminal sialic acid residue α2,6-linked to the galactose residue. Thus, Gal-3 binding to cell surface glycoproteins, including receptors, is normally blocked by sialylation of those glycoproteins, and is revealed by desialylation (Zhuo and Bellis, 2011; Nomura et al., 2017) (Figure 3).

Galectin-3 is highly expressed in myeloid, epithelial and endothelial cells, and fibroblasts. Within the cell, Gal-3 can be found in cytoplasm, nucleus, and membranes, and it can be released from cells following inflammatory stimuli such as LPS. Within the brain, Gal-3 is expressed by microglia, some astrocytes, and weakly by some cortical neurons (Yoo et al., 2017). Gal-3 expression is upregulated in neurodegenerative disease models (Siew et al., 2019; Boza-Serrano et al., 2019). One variant of the Gal-3 gene (LGALS3) is associated with Parkinson’s disease risk at *p* = 9 × 10^–15^ (Pickrell et al., 2016) and 4 × 10^–16^ (Chang et al., 2017). Gal-3 gene variants are also weakly associated with AD (Boza-Serrano et al., 2019).

We and others have shown that LPS-activated microglia release Gal-3 (Burguillos et al., 2015; Nomura et al., 2017). Gal-3 lacks an endoplasmic reticulum-targeting sequence, and therefore does not follow the classical pathway via endoplasmic reticulum and Golgi out of the cell. The mechanism of Gal-3 release from the cytoplasm is unclear, but it appears to be triggered by a rise in cytosolic calcium (Liu et al., 1995). We found that inhibition of calcineurin (a calcium-activated protein phosphatase) blocked LPS-induced Gal-3 release from microglia, suggesting the possibility that dephosphorylation of Gal-3 regulates its release (Nomura et al., 2017). Gal-3 can be phosphorylated on Ser6 and Ser12, which regulates localization and oligomerization. Extracellular Gal-3 can bind to multiple components of the extracellular matrix, and mediate interactions between cells and the extracellular matrix (Dumic et al., 2006).

Extracellular or intracellular Gal-3 levels are elevated in a variety of pathologies, potentially due to neuroinflammation. Extracellular Gal-3 levels were higher in CSF of AD patients (Ashraf and Baeesa, 2018). Amyotrophic lateral sclerosis patients (Ashraf and Baeesa, 2018) after birth asphyxia in humans (Savman et al., 2013) and after brain trauma in mice (Yip et al., 2017). Brain levels of Gal-3 were higher after brain ischemia (Yan et al., 2009) and in Huntington’s disease patients and a mouse model of Huntington’s disease (Siew et al., 2019).

Galectin-3 promotes neuroinflammation by multiple mechanisms (Shin, 2013; Chen et al., 2014). Extracellular Gal-3 can activate microglia apparently by directly activating TLR4 (Burguillos et al., 2015) and can thereby induce neuroinflammation after brain trauma (Yip et al., 2017) and brain ischemia (Rahimian et al., 2018). Consequently, lack of Gal-3 attenuates neuroinflammation, for example, in the retina and optic nerve of diabetic mice (Mendonca et al., 2018). Gal-3 knockout reduced microglial activation in response to brain ischemia in mice (Lalancette-Hebert et al., 2012). And Gal-3 knockout or inhibition reduced microglial activation in response to α-synuclein in culture (Boza-Serrano et al., 2019).

Extracellular Gal-3 can act as an opsonin (Nomura et al., 2017), i.e., it can bind to a cell’s surface and then induce phagocytes to phagocytose that cell by also binding and activating a phagocytic receptor on the phagocyte (Figure 3). Gal-3 binds and activates the phagocytic receptor MerTK (Caberoy et al., 2012) thereby inducing the phagocytosis of Gal-3 opsonized cells, debris, and aggregates by MerTK-expressing phagocytes (Nomura et al., 2017). Moreover, Gal-3 can bind to bacteria and therefore opsonizes bacteria for phagocytosis by microglia (Abreu et al., 2017; Cockram et al., 2019). Extracellular Gal-3 binds to desialylated cells and therefore opsonizes desialylated cells for phagocytosis (Nomura et al., 2017). Gal-3 is released after brain trauma in mice, and increases subsequent neuronal loss, so Gal-3 knockout or anti-Gal-3 antibodies reduce neuronal loss and brain damage (Yip et al., 2017). Similarly, Gal-3 increases after neonatal brain ischemia, and Gal-3–knockout mice are protected from the subsequent neuronal loss (Doverhag et al., 2010). Again, in optic nerve injury, Gal-3 knockout reduced retinal ganglion cell loss (Abreu et al., 2017). In each case, extracellular Gal-3 might promote neuronal loss by opsonizing neurons or by activating microglia—it is not clear which.

Galectin-3 enhances microglial phagocytosis of myelin, which may contribute to myelin-debris clearance (Rotshenker et al., 2008; Rotshenker, 2009). By contrast, extracellular Gal-3, released from microglia, can promote oligodendrocyte differentiation, so Gal-3 knockout mice have reduced axon myelination (Pasquini et al., 2011; Thomas and Pasquini, 2018). However, Gal-3 promotes neuroinflammation in experimental autoimmune encephalomyelitis (EAE) mouse models of multiple sclerosis (MS), so Gal-3 knockout reduces the severity of EAE (Jiang et al., 2009).

We found that Gal-3 was highly upregulated in the brains of AD patients and 5xFAD mice, a mouse model of AD, and this increase was found specifically in the microglia associated with amyloid plaques (Boza-Serrano et al., 2019). Importantly, Gal-3 knockout in 5xFAD mice display reduced microglial expression of pro-inflammatory genes *in vivo*, decreased amyloid plaques, and improved cognitive performance (Boza-Serrano et al., 2019). In agreement with its detrimental role in AD, co-injection of Gal-3 and Aβ was found to increase amyloid plaque deposition. Gal-3 associated with microglial TREM2 *in vivo* and bound to pure TREM2 *in vitro* (Boza-Serrano et al., 2019). Tao et al. (2020) found remarkably similar results: Gal-3 expression was upregulated in the brains of AD patients and APP/PS1 mice, another mouse model of AD; Gal-3 promoted Aβ oligomerization and fibrilization; Gal-3 knockout in APP/PS1 mice reduced amyloid plaque formation and decreased cognitive deficits; and Gal-3 bound microglial TREM2 to induce microglial activation and further Gal-3 expression. Thus, it appears clear that Gal-3 promotes amyloid pathology by amyloid aggregation, whether this has anything to do with the binding to TREM2 is less clear.

## Conclusion

Overall, these studies indicate that Gal-3 and sialylation/desialylation are important for neuroinflammation and potentially neurodegeneration. However, several questions remain open, and require further research. 1. Does desialylation of microglia, neurons, or synapses occur in neurodegeneration, and if so, when, and where? 2. Is complement deposition and microglial phagocytosis of synapses mediated by desialylation of synapses? 3. Does inhibition of extracellular sialidases prevent neurodegeneration? 4. Does inhibition of Gal-3 prevent neurodegeneration?

## Author Contributions

The authors contributed equally to writing this review.

## Conflict of Interest

The authors declare that the research was conducted in the absence of any commercial or financial relationships that could be construed as a potential conflict of interest.
